# Fibrocytes: A Novel Stromal Cells to Regulate Resistance to Anti-Angiogenic Therapy and Cancer Progression

**DOI:** 10.3390/ijms19010098

**Published:** 2017-12-29

**Authors:** Hisatsugu Goto, Yasuhiko Nishioka

**Affiliations:** Department of Respiratory Medicine and Rheumatology, Graduate School of Biomedical Sciences, Tokushima University, 3-18-15 Kuramoto-cho, Tokushima 770-8503, Japan; hgoto@tokushima-u.ac.jp

**Keywords:** cancer, angiogenesis, anti-angiogenic therapy, resistance, tumor stroma, fibrocyte

## Abstract

An adequate blood supply is essential for cancer cells to survive and grow; thus, the concept of inhibiting tumor angiogenesis has been applied to cancer therapy, and several drugs are already in clinical use. It has been shown that treatment with those anti-angiogenic drugs improved the response rate and prolonged the survival of patients with various types of cancer; however, it is also true that the effect was mostly limited. Currently, the disappointing clinical results are explained by the existence of intrinsic or acquired resistance to the therapy mediated by both tumor cells and stromal cells. This article reviews the mechanisms of resistance mediated by stromal cells such as endothelial cells, pericytes, fibroblasts and myeloid cells, with an emphasis on fibrocytes, which were recently identified as the cell type responsible for regulating acquired resistance to anti-angiogenic therapy. In addition, the other emerging role of fibrocytes as mediator-producing cells in tumor progression is discussed.

## 1. Introduction

In 1787, the term “angiogenesis” was originally introduced by the British surgeon John Hunter to describe the formation of new vessels in the process of wound healing [1]. Angiogenesis is an essential process of forming new vessels from existing vasculature in order to maintain the delivery of oxygen to a certain tissue and to remove carbon dioxide and waste products [2]. Almost two centuries after this term was proposed, it was suggested that this process of angiogenesis was also crucial to the survival and growth of tumor cells [3]. Since then, the field of angiogenesis research has rapidly expanded, and many different angiogenic and angiostatic factors and pathways have been identified as therapeutic targets [4,5,6]. Indeed, numerous angiogenesis inhibitors have been developed, and some of them are already clinically approved for cancer treatment [7]. For instance, the effect of bevacizumab, a first-approved monoclonal antibody that inhibits vascular endothelial growth factor (VEGF), was shown by phase III clinical trials to improve the response rate and survival of patients with non-small cell lung cancer (NSCLC) and colon cancer [8,9]. Currently, in addition to bevacizumab, a number of anti-angiogenic agents (i.e., sunitinib, sorafenib and ramucirumab) are in clinical use, and most are recognized as standard treatment options for many types of cancer.

One of the early motivations for developing anti-angiogenic agents was the hope that resistance to these drugs would not develop because their target was the genetically stable host endothelial cells [10,11]. However, subsequent clinical experience revealed that a significant number of cancer patients either do not respond to anti-angiogenic agents or develop resistance to them after an initial response [12,13]. Indeed, in 2011, an announcement was made by the US Food and Drug Administration (FDA) revoking the approval of bevacizumab for the treatment of metastatic breast cancer due to insufficient efficacy and safety [14]. This suggests the existence of mechanism(s) of resistance against anti-angiogenic drugs and that biomarkers for the efficacy of anti-angiogenic drugs (or resistance to them) are lacking. Both intrinsic and acquired resistance are now considered to be major factors that contribute to the limited clinical benefits of anti-angiogenic drugs [15].

A number of studies have been conducted to uncover the mechanism(s) of resistance to anti-angiogenic therapy; changes within the tumor cells seem to be the most intensively reported mechanism (Table 1). Because anti-angiogenic agents induce hypoxia inside the tumor via the suppression of new vessel formation, the tumor cells in this environment obtain the ability to express hypoxia inducible factor (HIF) and secrete multiple angiogenic growth factors. The production of growth factors other than those inhibited by anti-angiogenic drugs would allow tumor cells to induce re-angiogenesis and evade therapy [16,17,18]. Other modes of tumor cell-involved mechanisms of resistance include vasculogenic mimicry [19,20], vessel co-option [21,22] and the sequestration of drugs in intracellular vesicles [23,24,25,26,27]. A minor population of cancer cells even gives rise to pericytes to support the vessel function and tumor growth [28]. Tumor cells exploit one or more of these mechanisms to evade anti-angiogenic therapy.

In addition to the abovementioned tumor cell-induced resistance mechanisms, it has also become evident that several extrinsic mechanisms are involved in resistance to anti-angiogenic therapy. Most of these mechanisms take place within the tumor stroma, which consists of various host cells including fibroblasts, myeloid cells, pericytes and endothelial cells [5,16,29]. The importance of these stromal cells in tumor growth has been intensively studied as stromal cells can regulate tumor growth both positively and negatively, and these cells could be a potential therapeutic target. Consistently, it has become more apparent that these non-cancerous cells not only regulate tumor growth but also play an important role in resistance to anti-angiogenic therapy [16,29,30,31].

For instance, stromal cells (and tumor cells) secrete the variety of angiogenic factors such as platelet-derived growth factor (PDGF), placenta growth factor (PlGF), fibroblast growth factor (FGF), insulin-like growth factor (IGF) and angiopoietin-2 in addition to VEGF. In the tumor microenvironment, stromal cells and tumor cells form complicated network via these soluble factors in addition to the direct contact to initiate the resistance to anti-angiogenic therapy [5,16,29]. Taken together, the resistance to anti-angiogenic therapy is regulated by diverse mechanisms, including those related to the stromal and tumor cells, although their respective functions remain incompletely understood.

With the hypothesis that there are still uncovered stromal cell-induced molecular and/or cellular mechanisms that regulate resistance to anti-angiogenic therapy, we conducted the series of studies using mouse models and human lung cancer clinical specimens resected from patients after anti-VEGF therapy, and recently identified bone marrow-derived fibrocytes, which are double-positive for α-1 type I collagen and C–X–C chemokine receptor type 4 (CXCR), as a previously unrecognized stromal cell type involved in the acquired resistance to anti-angiogenic therapy. Fibrocytes were found to be involved in the network of the resistance mechanisms by producing FGF2. 

In this review, we describe various stromal cell types, including fibrocytes, that contribute to the mechanism of resistance to anti-angiogenic therapy, and discuss other emerging roles of fibrocytes in the regulation of cancer growth.

## 2. Stromal Cells Are Involved in the Resistance to VEGF Blockade

### 2.1. Endothelial Cells

The endothelial cells that line the luminal side of the blood vessels are the main target for anti-angiogenic therapy. As stated above, it has been considered that targeting endothelial cells are logically favorable in terms of drug resistance because these cells are genetically stable. However, recent studies have indicated that there are several mechanisms by which endothelial cells mediate resistance to anti-angiogenic therapy. Akiyama et al. reported that the endothelial cells express drug efflux pumps, such as P-glycoprotein and breast cancer resistance protein, to decrease intracellular drug concentration [32,33]. Moreover, Croci et al. recently reported that there is an endothelial cell-specific compensatory mechanism regulated by glycosylation-dependent lectin-receptor interactions to maintain VEGF signaling in the absence of ligand-receptor binding [34]. Considering the origin of tumor endothelial cells, Wang et al. reported that a certain population of cancer cells differentiates into tumor endothelial cells, and that VEGF blockers can only partially inhibit this process [35]. These results indicate that the endothelial cells do mediate the resistance (possibly intrinsic resistance) to anti-angiogenic therapy, independent from the alteration of the tumor cell-status and growth factor redundancy.

### 2.2. Pericytes

Pericytes are the cells that physically cover the blood vessels to protect endothelial cells and modulate the blood flow, vessel structure and permeability [36,37,38]. While normal blood vessels are covered with a layer of pericytes, the abnormal blood vessels in a tumor tend to have decreased pericyte coverage [39]; thus, the tumor vasculature is unstable and leaky, and can be sensitive to anti-angiogenic therapy. When tumors are treated with anti-angiogenic drugs, the pericyte coverage of the remaining blood vessels increases [40], suggesting that some of the vessels that are covered with pericytes are protected from anti-angiogenic drugs and that these vessels may initiate the acquired drug resistance. As a protective mechanism, pericytes influence the negative regulation of endothelial cell proliferation, rendering them quiescent and less sensitive to anti-angiogenic drugs [41]. Furthermore, Cascone et al. reported that increased activated epidermal growth factor receptor (EGFR) was detected on the pericytes of xenografts that acquired resistance to anti-angiogenic therapy, suggesting that gene instability may occur in the pericytes in the tumor [42]. The increased pericyte coverage in the remaining vessels after anti-angiogenic treatment could be the result of pruning of the neovasculature. However, it has also been reported that a minor population (cancer stem cells) of tumor cells have the potential to generate pericytes [28]. Collectively, these findings suggest that in addition to their role in the development of acquired resistance to anti-angiogenic therapy, pericytes may also mediate intrinsic resistance.

### 2.3. Tumor-Associated Macrophages

Among the myeloid-derived cells, tumor-associated macrophages (TAMs) seem to be the most intensively studied cell type to contribute not only to the progression of cancer but also to resistance to anti-angiogenic therapy. The origin of TAMs is considered to be circulating monocytes, and various chemoattractants, such as C–C motif chemokine ligand 2 (CCL2) or VEGF—which are produced by tumor tissue—lead monocytes to infiltrate into the tumor [43,44,45]. During this process, monocytes are known to differentiate into two major phenotypes: M1 and M2 macrophages [46,47]. This phenotypic plasticity of macrophages was originally identified in order to maintain immune and inflammatory homeostasis in tissue [48,49]. Subsequently, the plasticity of macrophages has received attention in the field of cancer biology, as the balance of M1/M2 is lost in the tumor tissue, which becomes M2-dominant [50,51,52]. These TAMs mediate the resistance to anti-angiogenic therapy by producing pro-angiogenic factors, especially in the hypoxic environment induced by the initial angiostatic treatment [47,51,53,54]. Moreover, TAMs also produce a variety of matrix metalloproteinases (MMPs) to degrade the extracellular matrix, which results in expanding of the space for the tumor cells and endothelial cells to proliferate and migrate [55,56,57,58]. Within M2 macrophages, the subpopulation expressing TIE2, namely TIE2-expressing macrophages (TEMs), is reported to be particularly highly pro-angiogenic [59,60], and to also contribute to resistance against anti-angiogenic therapy [61].

### 2.4. Myeloid-Derived Suppressor Cells

Under normal conditions, the immature myeloid cells that reside in the bone marrow differentiate into mature cells such as macrophages, neutrophils or dendritic cells and lose their original immunosuppressive feature when they infiltrate into peripheral blood or tissue [62,63,64]. However, under pathological conditions, these immature myeloid cells remain in an immature state and become highly immunosuppressive. In addition to their immunosuppressive activity, these myeloid-derived suppressor cells (MDSCs) play important roles in tumor progression and angiogenesis [62,65,66,67,68]. Regarding the role of MDSCs in the resistance to anti-angiogenic therapy, it was reported that tumors refractory to VEGF blockade contained increased numbers of tumor-infiltrating CD11b^+^Gr-1^+^ MDSCs in mouse models [69,70]. Moreover, these MDSCs promote tumor growth regardless of whether anti-VEGF antibody treatment is administered, through their production of VEGF and Bv8 [69,70]. One should note that the MDSCs in mice are defined as CD11b^+^Gr-1^+^ cells, while the definition of MDSCs in humans is still under debate [71,72].

### 2.5. Cancer-Associated Fibroblasts

Cancer-associated fibroblasts (CAFs) are one of the major stromal cells in tumors and play important roles in various aspects of cancer progression, such as tissue remodeling with the production of extracellular matrix, angiogenesis, cell invasion and therapeutic resistance [73,74,75,76,77]. With regard to the mechanism through which CAFs regulate resistance to anti-angiogenic therapy, it has been reported that, in addition to the variety of factors that are produced by CAFs, CAFs express CXCL12, PDGF-C and CD44 in tumors refractory to anti-angiogenic therapy, and that these factors recruit endothelial progenitor cells, induce a compensatory pro-angiogenic mechanism, or promote the stemness of cancer cells [78,79,80].

### 2.6. Other Stromal Cells

The resistance to anti-angiogenic therapy may be regulated by other types of stromal cells (Table 1). For instance, Chung et al. recently demonstrated that interleukin-17 (IL-17) produced by T helper type 17 (T_H_17) cells promotes tumor resistance to the inhibition of VEGF by mediating immature myeloid-cell mobilization and recruitment into the tumor microenvironment [81]. Platelets are known to scavenge anti-angiogenic drugs, such as bevacizumab and sunitinib, which affect the pharmacodynamics and bioavailability of these drugs [82,83]. Tumor-associated neutrophils (TANs), eosinophils, mast cells and dendritic cells might also have the potential to mediate the resistance to anti-angiogenic therapy; however, there is currently no direct evidence to support the involvement of these cells in the mechanism of resistance. Because they produce various chemical mediators that stimulate angiogenesis [84,85,86,87,88,89], it is possible that they might somehow be involved in resistance to anti-angiogenic therapy. Further studies should be performed to investigate the possible mechanism of resistance mediated by these stromal cells.

## 3. The Role of Fibrocytes in Re-Angiogenesis after VEGF Blockade

In addition to the abovementioned stromal cells that contribute to the resistance to anti-angiogenic therapy, we recently identified bone marrow-derived fibrocytes—which are double-positive for α-1 type I collagen and CXCR4—as a previously unrecognized cell type involved in the acquired resistance to VEGF blockade [90].

Fibrocytes, which are present in the peripheral circulation as a minor population of leukocytes, were first identified more than a decade ago [91]. Subsequent studies revealed that they are monocyte-derived cells that have the features of both macrophages and fibroblasts [92,93,94,95,96]. Since there is currently no single specific marker for fibrocytes, the combination of intracellular collagen staining and the expression of a hematopoietic marker, such as CD45, plus either CD34 or CXCR4, has been considered to be a sufficiently accurate criterion for identifying fibrocytes [92,93,97]. Based on their fibrogenic properties, fibrocytes were previously reported to be involved in the pathogenesis of various fibrotic diseases, such as pulmonary fibrosis, bronchial asthma and cardiovascular disease [98,99,100,101,102]. In the field of cancer, however, only a small number of studies have reported the existence of fibrocytes in the peripheral blood of patients [103], and the role of fibrocytes in the pathogenesis of cancer is unknown.

**Figure 1:**
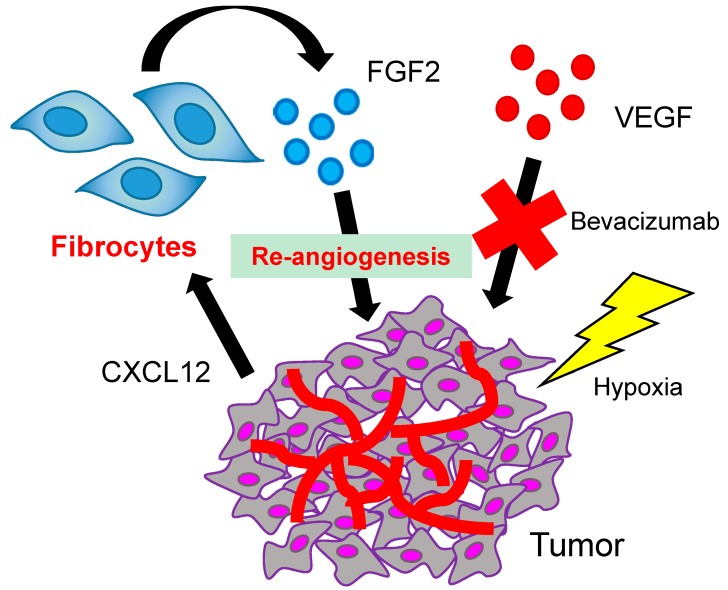
The possible role of fibrocytes in resistance to anti-angiogenic therapy. The hypoxic condition resulting from bevacizumab treatment could lead tumor cells to produce CXCL12, which would in turn prompt the fibrocytes to migrate into the tumor and produce FGF2.

We previously reported the efficacy of the single use of bevacizumab or the combination use of bevacizumab and conventional cytotoxic drugs in slowing the progression of human malignant pleural mesothelioma cells in immunodeficient mice [104,105]. In these studies, treatment with bevacizumab successfully prolonged the survival of mice, although the treatment did not completely suppress the tumor growth. These results led us to consider that there might be a mechanism of resistance underlying the VEGF blockade. In mouse models of lung cancer and malignant mesothelioma, we confirmed that mice became moribund due to tumor progression, despite continuous bevacizumab treatment [90]. To investigate whether this resistance to bevacizumab was intrinsic or acquired, tumor sections from different time points were subjected to CD31 staining. As a result, the microvessel density was found to be significantly reduced by bevacizumab treatment; however, it gradually increased over time with continuous bevacizumab treatment, suggesting that the resistance to bevacizumab in these tumors was acquired. A comprehensive analysis revealed that host cell-derived FGF2 was up-regulated in the bevacizumab-resistant tumor, suggesting that FGF2 played an important role in the acquired resistance. The crucial role of FGF2 was confirmed by the combination use of bevacizumab and FGF receptor inhibitor (BGJ-398) or anti-FGF2 antibody, and the combination therapy partially overcame the resistance to anti-VEGF therapy in mice. We performed flow cytometry and immunohistochemistry to identify the stromal cells that produce FGF2, and identified that FGF2 was produced by CD45^+^CXCR4^+^collagen type I^+^ cells, which are known as fibrocytes. Of note, a similar result was obtained in a syngeneic mouse model using B16 melanoma cells and C57BL/6 mice treated with SU5416, a VEGF receptor inhibitor. A subsequent in vitro experiment confirmed that the culture supernatant of fibrocytes enhanced the proliferation of endothelial cells, which was blocked by BGJ-398. These results suggested that the fibrocytes play, at least in part, an important role in resistance to anti-VEGF therapy as a producer of FGF2 in addition to other stromal cells such as fibroblasts and macrophages. In order to determine the mechanism by which fibrocytes were recruited into the tumor environment, we performed human-specific gene profiling using tumor tissues, and found that C–X–C motif chemokine ligand 12 (CXCL12), the ligand of CXCR4, played a role in the recruitment of fibrocytes. Because CXCL12 is the target molecule of HIF-1α, the hypoxic condition resulting from bevacizumab treatment could lead the tumor cells to produce CXCL12, which would in turn prompt the CXCR4^+^ cells to migrate into the tumor (Figure 1). As others reported, this mechanism seems to be commonly involved in the recruitment of other cell types such as TAMs and endothelial progenitor cells [106,107]. More importantly, fibrocytes were detected in human lung cancer tissue and the number of tumor-infiltrating fibrocytes was correlated with the use of bevacizumab prior to the surgery. The number of fibrocytes was significantly higher in tumors from patients who received bevacizumab-containing chemotherapy prior to the surgery in comparison to the tumors from patients who received chemotherapy alone or who received no prior therapy. Of note, the number of fibrocytes in the bevacizumab-treated patients was significantly correlated with the number of bevacizumab treatment cycles and with the vessel length in the tumor. Taken together with the results from mouse models, these findings indicated that fibrocytes may be one of the key regulatory cells in the tumor microenvironment that are involved in the acquisition of resistance to anti-VEGF therapy through their production of FGF2.

## 4. Other Emerging Roles of Fibrocytes in the Tumor Microenvironment

As stated above, fibrocytes are known as collagen-producing cells; thus, the majority of the fibrocyte research has been conducted in the field of fibrotic diseases. On the other hand, our recent work suggests that the fibrocytes functioned as mediator-producing cells rather than collagen-producing cells [90,108]. Indeed, the amount of collagen produced by fibrocytes was much lower than that produced by fibroblasts. On the other hand, the expression of growth factors such as FGF2 and PDGF in fibrocytes was significantly higher than that in monocytes. From this point of view, fibrocytes can act as inflammatory cells and may play a number of crucial roles in various diseases. Thus, in the field of cancer biology, fibrocytes may modulate not only the mechanism of resistance against anti-VEGF therapy but also the mechanism of tumor progression. For example, our recent work suggested that: (1) the number of infiltrating fibrocytes in human lung cancer tissue was correlated with poor survival of the patients; (2) fibrocytes enhanced tumor initiation in mouse xenografts; and (3) fibrocytes induce the stemness of cancer cells via multiple soluble factors [109]. These findings show that fibrocytes have diverse functions in the regulation of tumor progression as well as in the modulation of resistance to anti-angiogenic therapy.

## 5. Conclusions

Recent efforts to verify the mechanisms by which the stromal cells regulate the resistance to anti-angiogenic therapy have allowed us to uncover various cell types and signaling pathways. These stromal cells could potentially represent a target for therapy to overcome resistance as well as a biomarker of the efficacy of anti-angiogenic therapy, or conversely, a biomarker of refractoriness. The soluble factors present in the peripheral blood, such as VEGF, PlGF, CXCL12 and FGF2, which may be able to serve as biomarkers for resistance to anti-angiogenic therapy, have also been intensively studied [110,111,112,113,114,115]. Considering that these soluble factors have not been successfully developed as practical biomarkers in the clinical setting, and because the cell–cell interactions play crucial roles in the tumor microenvironment, it seems crucial to focus on cells and tissues at this point in time. Although it is challenging to obtain tumor tissues after treatment with anti-angiogenic drugs, given the small number of such samples available, continuous efforts are warranted to develop the technology that allows for the easy detection of tumors and stromal cells (i.e., via peripheral blood tests). Understanding the mechanism through which stromal cells mediate resistance in the tumor would help to improve the efficacy and durability of anti-angiogenesis therapy.

## Figures and Tables

**Table 1:** The list of tumor cell-mediated mechanisms and stromal cell types involved in the resistance to anti-angiogenic therapy.

Tumor Cell-Mediated Mechanisms	Stromal Cells Involved	Cells Possibly Involved
Growth factor redundancy Vascular mimicry Vessel co-option Vessel intussusception Intracellular drug sequestration Induction of stemness Endothelial cell differentiation Pericyte differentiation	Endothelial cells (including progenitor cells) TAMs (including TEMs) MDSCs CAFs Pericytes Platelets Lymphoid cells Fibrocytes	TANs Eosinophils Mast cells Dendritic cells

Note that tumor cell-mediated and stromal cell-mediated mechanisms are closely associated with the development of the actual resistance. TAMs, tumor-associated macrophages; TEMs, TIE2-expressing macrophages; MDSCs, myeloid-derived suppressor cells; CAFs, cancer-associated fibroblasts; TANs, tumor-associated neutrophils.
